# Continuous observation of Parkinsonian symptoms using symptom diaries & wearable accelerometry

**DOI:** 10.1038/s41597-026-06999-6

**Published:** 2026-04-09

**Authors:** T. P. R. Nesser, C. van der Linden, C. Schedlich-Teufer, G. Brandt, M. T. Barbe, T. A. Dembek

**Affiliations:** 1 University of Cologne, Faculty of Medicine, Department of Neurology, Cologne, Germany; 2 Charité Universitätsmedizin Berlin, Department of Neurology, Berlin, Germany

**Keywords:** Parkinson's disease, Neurological manifestations

## Abstract

Treatment adjustments in Parkinson’s Disease (PD) are often based on clinical evaluations at single time points which are insufficient to adequately assess real-life motor fluctuations. Patient-written symptom diaries on the other hand are highly subjective and require well-educated and adherent patients to provide reliable results. Wearable accelerometry might provide a reliable, objective, and continuous diagnostic method to assess PD motor symptoms & fluctuations. However, large datasets of simultaneous sensor data and symptom diaries are needed for such method development and validation. We here provide a well-described, open-science dataset of simultaneous, bilateral, wrist-worn accelerometry and symptom diary data from 66 participants (41 male, 25 female) with PD. On average, participants provided data for 6.0 consecutive days resulting in a total of 393.8 days for the dataset as a whole. Symptom diaries include data on kinetic state, tremor, freezing of gait, falls, and PD-related medication intake. Further demographic information is also provided. This dataset will support the development and validation of accelerometry-based approaches to assessing motor symptoms and fluctuations in PD.

## Background & Summary

Parkinson’s disease (PD) is the second most common neurodegenerative disease, causing a variety of motor and non-motor symptoms and severely impacting patients’ activities of daily living and quality of life. PD’s clinical hallmark is a characteristic movement disorder with bradykinesia, rigidity, and resting tremor of 4–6 Hz frequency^1^. While dopaminergic medication can lead to profound improvements, motor fluctuations with akinetic and dyskinetic phases are pathognomonic of advanced disease stages. Many PD medications, such as long-lasting dopamine-agonists, extended-release formulations of levodopa, or catechol-o-methyltransferase inhibitors as well as device-aided therapies such as deep brain stimulation (DBS), or subcutaneous pumps for apomorphine and foslevodopa were specifically designed to address these motor fluctuations. While motor fluctuations thus are of great importance in advanced PD stages and constitute one of the main targets for therapeutic interventions, there is a relevant lack in objective assessments of these fluctuations^2^.

To this day, the gold standard for assessing real-life motor fluctuations is the documentation within a symptom diary, either written by patients themselves or by their caregivers. Such diaries present many major drawbacks: First, they require well-educated and highly adherent patients or caregivers to provide reliable results. Second, they either need to be filled out constantly – which impacts daily living – or are impacted by recall issues and diary fatigue^2–7^. Third, it is well known that many PD patients experience anosognosia or impaired self-awareness for different motor symptoms, especially dyskinesia and tremor^8–10^. These issues highlight the need for reliable, accurate, objective, and continuous diagnostic methods that can capture motor symptoms and fluctuations in PD patients under real-life conditions^11,12^.

Wearable accelerometry offers potential to address many of the symptom diaries’ limitations. Accelerometers are ubiquitous in current smartphones and smartwatches and have been widely used in studies of movement, including movement analysis in PD patients. Over the past decade, various approaches have investigated the use of wearable accelerometers to record PD motor symptoms in real-world settings, with the aim of providing new information and much-needed objectivity to the assessment of PD motor symptoms^13–17^. Systems such as the Personal KinetiGraph^TM^ (PKG) or smartwatch-based monitoring solutions have demonstrated feasibility and patient acceptability^13,14^. In addition, Powers *et al*. developed an outpatient monitoring system based on consumer electronics to act as a clinical decision support tool to improve medication dosage and thereby enable more accurate treatment decisions^14^. Fisher *et al*. furthermore demonstrated a patient preference for wearing sensors over completing symptom diaries^18^.

Nevertheless, major challenges remain. The majority of previous studies were performed on small, highly selected cohorts, and outcome measures varied considerably between studies^16,19,20^. Moreover, long-term compliance with wearable sensors in everyday life is still poorly understood^21^, and digital outcome measures require further validation in clinical populations^22^. As highlighted by Bloem *et al*., currently available solutions for the accelerometric monitoring of PD lack sufficient clinical validation and should therefore be used with caution^4^. Additionally, there is a lack of consensus on how wearable-derived metrics should be standardised and implemented in clinical practice^12,19,22^. In particular, comparisons against established reference standards, such as symptom diaries, are still rare and not included in available open-science datasets of accelerometry data in PD^15,23^.

This study aims to address this issue by providing a large, comprehensive, open science dataset of simultaneous bilateral accelerometry and symptom diary data. This dataset may serve to validate existing and develop novel paradigms to classify PD motor symptoms and fluctuations.

## Methods

### Study design

**Fig. 1:**
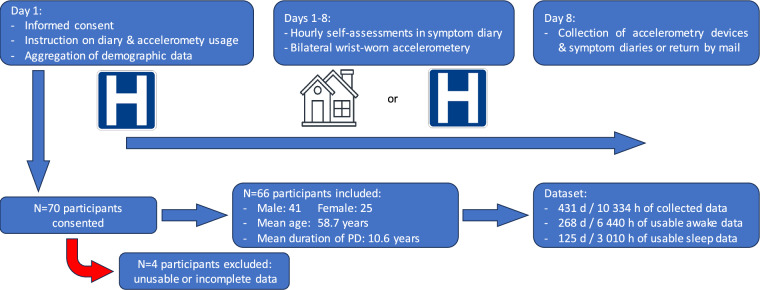
Illustration of the study protocol and recruitment numbers. [Abbreviation: H = hospital].

Our study on the continuous observation of Parkinsonian symptoms (COPS) was a single-centre, prospective, observational data acquisition study. The primary aim of the trial was to create an open-science dataset of simultaneous symptom diaries and bilateral wrist-worn, three-axis accelerometry, as well as supporting demographic and clinical information. To achieve this goal, participants were asked to wear three-axis-accelerometry devices on both wrists for up to 7 days in both in- and outpatient settings while filling out symptom diaries in hourly intervals. At the end of the study period, diaries and devices were collected from inpatients or received back by mail from outpatients. The study was approved by the institutional review board of the University of Cologne (Vote: 21-1569) and registered in the German Clinical Trials Register (DRKS00028636). The study was conducted in accordance with the Declaration of Helsinki and all participants provided written informed-consent prior to study participation. Data was collected between September 2022 and June 2023. Figure 1 provides an illustrated summary of the trial protocol, the recruitment numbers and some of the key data.

### Inclusion criteria

The inclusion criteria were a diagnosis of PD according to the medical report, the ability to provide written informed-consent, and being over 18 years of age. No further inclusion or exclusion criteria were specified, which enabled us to recruit a wide range of participants with PD. All participants were recruited from the in- and/or outpatient clinic at the Department of Neurology at the University Hospital of Cologne, Germany.

### Demographic data

The following demographic and disease-related data (also see Table 2) was collected from participants and/or their patient-files: age, sex, PD-subtype, lateralization, duration of illness, UPDRS-III-scale, Hoehn- & Yahr stage and treatment with deep brain stimulation (DBS) of the subthalamic nucleus (STN). None of the participants received pump therapies.

### Symptom diaries

**Fig. 2:**
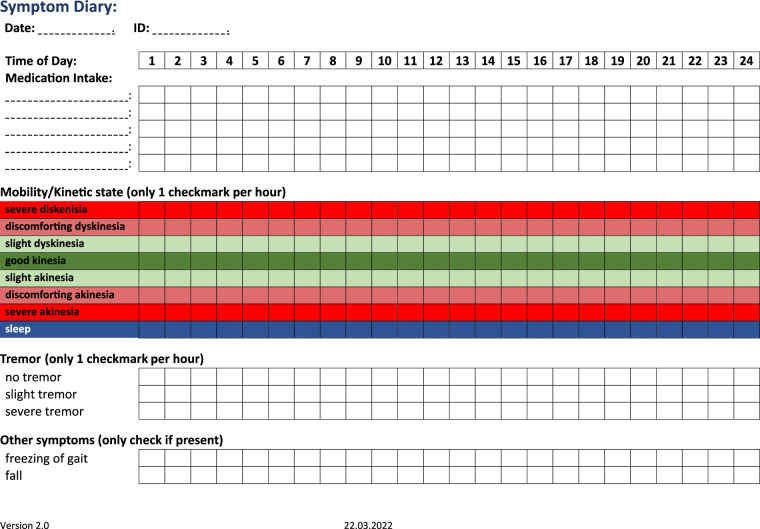
Symptom diary used for this study. Original files can be found within the dataset in pdf and docx format.

Participants were asked to fill out symptom diaries for seven consecutive days. Hourly self-assessments were recorded in diaries through tick marks representing the participants’ predominant status during the prior hour in terms of kinetic state (7-point scale), tremor (3-point scale), freezing of gait (binary scale), falls (binary scale), and timepoints of Parkinson-related medication intake. Furthermore, participants were asked to document their respective sleep schedule. All items of the symptom diary are described in detail in Table 1. A template of the diary in docx and pdf format can be found within the dataset and is shown in Fig. 2.

**Table 1:** Items of the symptom diary with examples & descriptions.

Hourly Symptom Diary			
Variable name	Variable content	Example	Description
ID	i.e. COPS – XX	COPS – 10	Pseudonym of the participant
Day	1 days – 7 days	Day5	The specific day of the recording period
Time	1 hr–24 hr	8 hr	The specific hour of the day
Visit	Inpatient – Outpatient – Mixed	Outpatient	Indicates whether on the respective day of recording the participant was in an inpatient, outpatient or mixed setting
Kinesia	Sleep – Severe Akinesia – Discomforting Akinesia – Slight Akinesia – Good Kinesia – Slight Dyskinesia – Discomforting Dyskinesia – Severe Dyskinesia	Slight Akinesia	Describes the participant’s movement condition, ranging from sleep to severe dyskinesia
KinesiaScore	0–7	3	Numerical score representing the severity of the participant’s movement condition
Tremor	No Tremor – Slight Tremor – Severe Tremor	No Tremor	Indicates the presence and severity of tremor
TremorScore	0–2	0	Numerical score representing the severity of the tremor
Freezing	No Freezing – Freezing	Freezing	Indicates whether the participant experienced freezing episodes
FreezingScore	0–1	1	Numerical score representing the occurrence of freezing
Fall	No Fall – Fall	No Fall	Indicates whether the participant experienced a fall
FallScore	0–1	0	Numerical score representing the occurrence of falls
Medication	[Name of medication]	Levodopa/Benserazid, Pramipexole ER	Names of medications taken by the participant
Dosage_X (one column per medication)	Numeric in [mg]	Dosage_1: 100	The dosage of Parkinson’s disease medications in milligrams
		Dosage_2: 3.15

### Accelerometry

Participants were asked to wear three-axis accelerometers on both wrists for seven consecutive days, while simultaneously completing the symptom diaries. GENEActiv (Activinsights, Kimbolton, United Kingdom) sensors were used throughout the study. These watch-style devices provide continuous three-axis accelerometry data as multiples of g (~9,81 m/s^2^) at a recording frequency of 100 Hz. Data is stored locally on the device and the available storage permits continuous recordings of up to one week. Moreover, the device records data from light and body temperature sensors, which provide additional information regarding whether the devices were worn at a given time. The device has been used extensively in medical and other research (also see https://activinsights.com/expertise/publications/). We deliberately chose an accelerometry-only device, due to the wide availability of three-axis-accelerometry and thus the enhanced generalizability of any potential results stemming from this dataset to other devices.

### Data processing

**Fig. 3:**
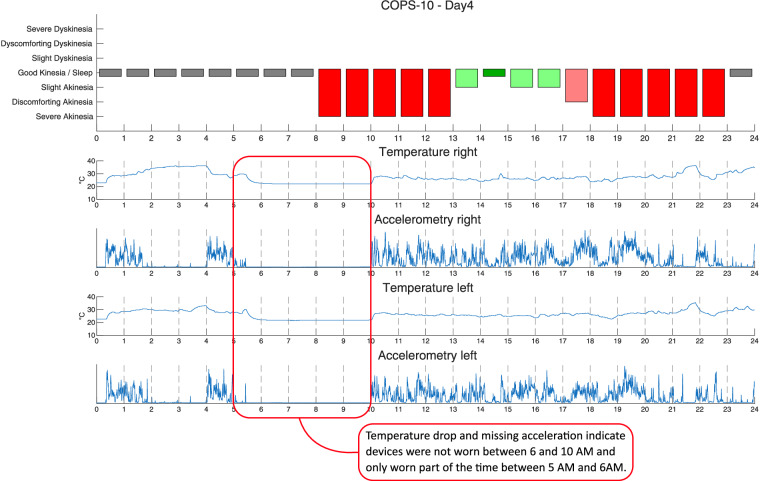
Example check for data plausibility for day 4 of the recordings of participant COPS-37: The temperature and movement profiles between 5:30 and 10:00 indicate that the sensors were not worn during this time. As a consequence, the corresponding data was categorised as “missing hours” and disregarded for subsequent analysis.

Paper diaries were digitized into Microsoft Excel (Microsoft, Redmond, USA) and then converted to csv format using custom Matlab (The Mathworks, Nattick, USA) scripts. Sensor data was extracted from the GENEActiv devices and converted into csv format using the accompanying software. All further processing and analyses were conducted using custom Matlab scripts. Diary and device data were combined by aligning timestamped device data with the symptom diaries. To allow for smaller file sizes and thus reduce computational demands, accelerometry data was split at one-hour intervals, which were stored as separate csv files and then compressed via the zip standard. All recorded data was checked for integrity, plausibility, and potential recording failures via visual inspection of summarizing figures (Fig. 3). If temperature values and corresponding movement profiles indicated that the watch had not been worn by the participant, corresponding data points were labelled accordingly within the symptom diary (item ‘WearableDataAvailability’, see Table 1).

## Data Records

**Fig. 4:**
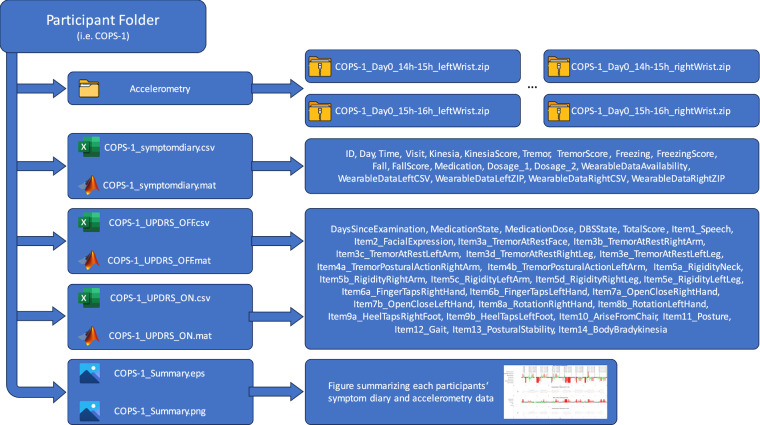
Data structure within the folder for each participant: Each folder contains a subfolder with the accelerometry data in zip-compressed csv-format – which was split on a per hour basis. Furthermore, each folder holds csv- and mat files containing (1) the symptom diary, (2) the UPDRS-III score in the medication off state, (3) the UPDRS-III score in the medication on state. Lastly, each folder contains a graphic summary of the contents of the symptom diary as well as the accelerometry data in png and eps format (see also Fig. 5).

The dataset can be accessed via the Open Science Framework at https://osf.io/5xvwn/ or via the 10.17605/OSF.IO/5XVWN^24^. After moving to the ‘Files’ tab, demographic data for all participants can be found in the table ‘Demographics’, both in mat and csv format. The ‘Data’ folder contains a single zip-file for each participant which can be downloaded and extracted at will. The resulting folder for each participant contains the symptom diary (e.g. ‘COPS-X_symtomdiary.csv’) and available UPDRS III scores (e.g. ‘COPS-X_UPDRS_OFF.csv’) in both mat and csv format as well as the hourly accelerometry data within the subfolder ‘Accelerometry’ in zip compressed csv-format. An overview of the folder structure is provided in Fig. 4.

**Fig. 5:**
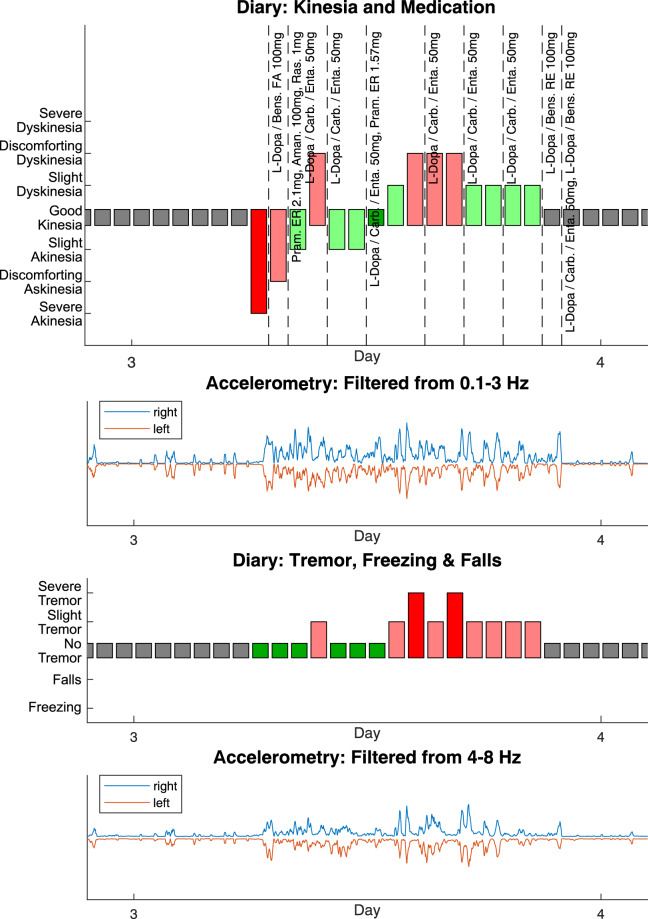
Summary figure for participant COPS-29, day 3. Upper panel: Kinesia state (red to green bars), sleep (grey bars), and medication intake. Second panel: Accelerometry data for right (blue) and left (orange) arm. Accelerometry data was summed over moving 5 minute intervals and filtered between 0.1–3 Hz. Third panel: Tremor severity (red to green bars) as well as freezing or fall events (not present in this participant). Bottom panel: Accelerometry data for right (blue) and left (orange) arm. Accelerometry data was summed over moving 5 minute intervals and bandpass-filtered between 4 Hz to 8 Hz to reflect tremor-related activity.

A detailed description of all items of the symptom diary can be found in Table 1. For UPDRS III scores the total score as well as all individual items are provided. Additionally, the files include information about how many days before study participation the UPDRS-III score was assessed (item ‘DaysSinceExamination’), whether the score was assessed after levodopa intake or without medication (items ‘MedicationState’ and ‘MedicationDose’), and/or whether DBS was turned on or off during the assessment (item ‘DBSState’). Accelerometry data csv files consist of the six columns ‘Time’, ‘X’, ‘Y’, ‘Z’, ‘Photo’, and ‘Temp’ which include the timestamp of the measeurement in milliseconds, the three accelerometry values of the different axes in multiples of g, a dimensionless value of the light sensor, and the temperature in degrees Celsius. Lastly, each participants’ folder includes a figure in png and eps format (e.g. ‘COPS-X_summary.eps’, see Fig. 5) which summarizes the contents of the symptom diary as well as an overview of the acceleration values. These figures allow to quickly examine how much data is available for a particular participant and whether the participant experienced a certain motor state like e.g. tremor or dyskinesia.

## Data Overview

A total of 70 participants were enrolled in the study. Four participants needed to be excluded from the dataset: Three participants were unable to correctly enter data into the symptom diary, while one participant accidentally destroyed their symptom diary. The final study population thus consisted of 66 participants (41 male, 25 female), with a mean age of 58.66 years (range: 46–76 years) and a mean disease duration of 10.6 years (range: 0–20 years). An overview of the demographic data can be found in Table 2.

**Table 2:** Demographic details of the study cohort.

Demographics				
Participants	66			
Mean [range] age	58.66 years [46–76 years]			
Sex	Male: 41		Female: 25	
Handedness	Left: 2		Right: 64	
PD subtype	Akinetic-rigid:	Equivalence-type:	Tremor-dominant:	
	24	21	21	
PD lateralization	Left: 24		Right: 42	
Mean [range] duration of PD	10.6 years [0–20 years]			
Hoehn & Yahr stages with respective number of participants	H&Y-1: 3	H&Y-1.5: 1	H&Y-2: 24	H&Y-2.5: 2
	H&Y-3: 31	H&Y-4: 5	H&Y-5: 0	
UPDRS-III – scale available	Yes: 64 (96.97%)		No: 2 (3.03%)	
STN – deep brain stimulation	Yes: 46 (69.70%)		No: 20 (30.3%)	

[Abbreviations: H&Y = Hoehn and Yahr, PD = Parkinson’s Disease, STN = subthalamic nucleus, UPDRS = Unified Parkinson’s Disease Rating Scale].

The dataset included a total of 430.6 days of collected data, with a median of 7.3 days (range: 1–8) per participant. Upon screening of the data, missing or implausible data was found for 4.2% of total accelerometry data (18.3 days) and 2.6% of total symptom diary data (11.3 days). Therefore, 393.8 days (91.5%) of simultaneous symptom diary and accelerometry data are available within in the dataset. Of the 393.8 days of usable data, 268.3 days were collected while participants were awake and 125.4 days while participants were asleep. The characteristics of the dataset can be found in Table 3.

**Table 3:** Characteristics of the dataset.

Dataset			
	Days	Hours	Percentage of Study Duration
Study Duration	430.6	10 334	—
Median time per Participant	7.3	174	—
Missing Symptom Diary	11.3	272	2.6%
Missing Accelerometry	18.3	438	4.2%
Missing Accelerometry (asleep)	4.1	99	0.96%
Missing Accelerometry (awake)	14.2	340	3.3%
Amount of Usable Data	393.8	9 450	91.5%
Amount of Usable Data (awake)	268.3	6 440	62.3%
Amount of Usable Data (asleep)	125.4	3 010	29.1%
Mean Amount of Usable Data per Participant	6	143.2	—
Median Amount of Usable Data per Participant	6.8	163.5	—

## Technical Validation

No technical validation was performed for this study since the GENEActiv device is a validated and widely used raw data accelerometry device^15,23,25–27^. In a previous study we compared the GENEActiv device against a BrainProductes 3-axis accelerometry device mounted at the tip of the index finger and found a comparable performance in the classification of resting tremor severity^17^.

## Data Availability

The full dataset can be downloaded via the Open Science Framework using the following link: https://osf.io/5xvwn/^24^.

## Code availability

Example code (Matlab, the Mathworks, USA) for data extraction and the generation of figures is included within the dataset in the subfolder ‘Scripts’.
